# Length of the period with late life dependency: Does the age of onset make a difference?

**DOI:** 10.1007/s10433-023-00777-8

**Published:** 2023-07-01

**Authors:** Susanne Kelfve, Jonas W. Wastesson, Bettina Meinow

**Affiliations:** 1 Department of Culture and Society, Linköping University, Norrköping, Sweden; 2 Department of Neurobiology, Care Sciences and Society, Aging Research Center, Karolinska Institutet & Stockholm University, Solna, Sweden; 3 Department of Medical Epidemiology and Biostatistics, Karolinska Institutet, Solna, Sweden; 4 Stockholm Gerontology Research Center, Stockholm, Sweden

**Keywords:** Last years of life, Fourth age, Long-term care, Gender differences, Living alone

## Abstract

There is a gap in knowledge about factors associated with the duration of late life dependency. In this study, we measured how the age at onset of late life dependency relates to the time spent with late life dependency. Using Swedish register data, we identified people 70 + who entered the period of late life dependency (measured by entering long-term care for help with PADLs) between June and December 2008. We followed this cohort (*n* = 17,515) for 7 years, or until death. We used Laplace regression models to estimate the median number of months with late life dependency by age group, gender, level of education and country of birth. We also calculated the crude percentiles (p10, p25, p50, p75 and p90) of month with late life dependency, by age group, gender and cohabitation status. Results show that the majority spent a rather long period with dependency, the median number of months were 40.0 (3.3 years) for women and 22.6 (1.9 year) for men. A higher age at entry was associated with a shorter duration of dependency, an association that was robust to adjustment for cohabiting at baseline, gender, education and country of birth. Our results suggest that older adults who postpone the start of dependency also compress the time with dependency, this lends support to the ambitions of public health initiatives and interventions targeting maintained independence in older adults.

## Introduction

Most older adults experience a period with functional dependency toward the end of life (Christensen et al. 2013; Gill et al. 2010; Romoren and Blekeseaune 2003; Smith et al. 2013; Zhao et al. 2010), a period often characterized by substantial care needs and use of long-term care (including homecare or residential care) (Meinow et al. 2020a). This period with dependency is sometimes referred to as the fourth age which can be contrasted with the third age, i.e., the part of life after retirement but without dependency (Baltes and Smith 2003; Laslett 1994). The length of the period with late life dependency, as well as the age at onset, has significant implications not only for the individual, but also for the society in providing healthcare and long-term care.

On a population level, it is difficult to monitor when individuals enter late life dependency, since population-based studies lack incidence data on dependency. In addition, no easily measured indicator for late life dependency in a population exists. From a societal perspective, the use of long-term care may be a relevant indicator of individuals' dependency, especially in countries where long-term care is publicly funded and used by all societal groups. In this paper, we use nationwide Swedish register data of long-term care use with an intention to capture late life dependency. This novel approach to approximate late life dependency is not without limitations but may be a complement to more time- and resource consuming approaches, such as longitudinal survey data, which is often limited by small samples and selective attrition (Chatfield et al. 2005; Kelfve et al. 2017).

The patterns of late life dependency and use of long-term care have mostly been studied in retrospective studies of decedents followed-back for one or two years from death (Gill et al. 2010; Meinow et al. 2020a; Zhao et al. 2010). Studies show that older age, female gender and severe dementia increase the likelihood of being severely disabled throughout 1–2 years before death (Gill et al. 2010; Smith et al. 2013; Zhao et al. 2010). However, the period with late life dependency can be substantially longer than 2 years and little is known about the length of the entire period with late life dependency and what factors that are related to the length of this period. In particular, little is known about how the age at which dependency starts relates to the duration of this period (until death).

On the one hand, a higher age at entrance could be related to a shorter period of dependency since higher age per se is associated with higher risk of mortality (Harman 2003; Wang et al. 2012). On the other hand, entering the fourth age at a higher age could also be associated with a longer duration of dependency, as a later entry may indicate a healthier aging process, that could be related to longer survival even after becoming dependent. To capture the entire period with late life dependency toward the end of life, prospective studies are necessary, but have rarely been conducted.

One notable example of a prospective study with detailed and long follow-up of late life dependency is a local Norwegian study, conducted from the 1980s to the 1990s. In this study, all persons 80 years and older in one municipality (*N* = 434) were followed until death (Romoren and Blekeseaune 2003). Results showed that few older adults experienced a sudden death (15% among men and 6% among women), whereas a majority, almost 80% among women and 55% among men, experienced dependency in personal activities of daily life (PADLs), such as the ability to eat, use the toilet, dress/undress and get up/go to bed, before death. Among those who experienced dependency in several PADLs at some timepoint before death, their total period with dependency lasted on average 4.9 years for women and 4.0 years for men, of which about half the time consisted of dependency in only one PADL (Romoren and Blekeseaune 2003).

The main drawback of this Norwegian study is the restriction to a small study sample in one municipality, addressing conditions several decades ago. We will address the length of the period with late life dependency using Swedish national and routinely collected administrative data of long-term care use among individuals aged 70 years or older. In Sweden, the use of long-term care has been shown to be a good proxy for late life dependency; a study among older adults in Stockholm showed that 70–90% of those dependent in PADLs used publicly financed long-term care (i.e., home care or residential care) (Lagergren et al. 2016).

One reason for the high agreement between long-term care use and dependency in Sweden is the universal long-term care system, that is largely financed by local taxes and available for all inhabitants aged 65+ . Need assessors employed by the municipalities assess individual care needs and decide about what kind and how much care an individual is entitled to. Since access is needs-tested, but not means-tested, services are used by all socioeconomic groups. Individual income-related user fees are low, with a cap of currently ~ $225 per month, covering 4% to 5% of the actual costs (Johansson and Schön 2017). Household or family economic resources are not considered.

Our objective is to analyze prospectively how the age at onset of dependency relates to the time spent with late life dependency. Using long-term care for help with PADLs as a proxy of late life dependency, we follow a nationwide cohort of people entering the late life dependency for 7 years.

## Method

### Population

Using individually linked nationwide Swedish registers, we identified people 70 years and older who entered the period of late life dependency between June and December 2008 and were still alive 31 December 2008. This cohort (*n* = 17,515) was followed for 7 years, until 31 of December 2015, or until death.

### Data source

Late life dependency was assessed using long-term care with PADLs as identified in the Social Services Register (SSR). SSR is a routinely collected administrative register, administrated by National Board of Health and welfare (NBHW), started in 2007 where information about the provision of publicly funded elderly care is included (reported by the municipalities). From 2007 to 2012, all municipalities were asked one or two times a year to report type and amount of long-term care for all inhabitants. Since 2013, the register includes monthly data. According to NBHW, the data quality in SSR is sufficient for statistical purposes with one exception (Meyer et al. 2022). The data from 2009 is not reliable to estimate the total amount of services on the population level, due to double reporting (both new and old services could be reported in the same month) for some individuals after a change in the report system. However, the problem is less problematic on an individual level, especially for residential care since almost no one leave residential care. In addition, there is also some variation regarding how the municipalities report specific types of services, some municipalities do not report hours of home care, but instead type of service (Meyer et al. 2022). Since this study is about to identify people entering the late life dependency period and not estimating amount of elder care services on a population level, we value these limitations in the data quality less problematic.

### Entrance of late life dependency

*Entrance of late life dependency* was defined as using long-term care with PADLs (defined as living in residential care or using homecare for help with PADLs or use of > 24 h of homecare per month) in December 2008, but not in an earlier data collection, i.e., June 2008 and October 2007.

The registers do not include the reason behind long-term care and individuals can enter the long-term care system for other reasons than dependency related to PADL. Institutional care is, however, a good proxy for dependency in PADL, since the threshold for institutional care is high in Sweden and not an option for individuals without dependency in PADLs. For homecare, some municipalities indicate that the home care service includes help with PADLs, others the number of hours with homecare. Since reporting routines differ between municipalities, we used either the type of homecare (help with PADLs) or the use of at least 25 h per month as an indicator of dependency in PADLs to avoid including those with only minor needs, such as cleaning and food boxes.

To be considered a debutant with late life dependency, people still alive also needed to use long-term care in PADLs at the remaining data collections 2009–2012. In this way, we tried to avoid the inclusion of people with short-term dependency. However, we accepted one missing data point during this period, to allow for administrative problems with reporting. By using a conservative strategy to identify the group of debutants, we do not capture all debutants but can instead be more confident that our study population consist of people entering late life dependency.

### Outcome

We measured *Duration of late life dependency* by months from 30 September 2008 (the mid date between 30 September and 31 December) until death or 31 December 2015. *Date of death* was collected from the National Cause of Death Register.

### Predictor

We calculated* age at start* as 2008 minus birth year and grouped it into 6 categories (70–74, 75–79, 80–84, 85–89, 90–94 and 95+).

### Covariates

*Cohabitation status* (cohabiting, living alone), *education* (compulsory, upper secondary, tertiary) and *country of birth* (Sweden, the Nordic region, outside the Nordic region) were collected from Statistics Sweden and refer to information from year 2008.

### Analyses

Due to the asymmetric distribution of the outcome variable, as well as the presence of censored cases, we used Laplace regression models to estimate the median months of late life dependency, by age group, gender, level of education and country of birth, bivariate as well as adjusted for all covariates. Laplace regression is a quantile regression model for longitudinal data that can handle both asymmetric and censored data (Bottai and Zhang 2010). We also calculated crude percentiles (p10, p25, p50, p75 and p90) of months with late life dependency, by age group, gender and cohabitation status. That is, for each analyzed group (e.g., cohabiting women 70–74 years old) we calculated the number of months until 10, 25, 50, 75 and 90 percent of the group had died.

Ethical approval for record-linkage of the Swedish register data was obtained from the Stockholm Regional Ethical Review Board (Dnr 2016/1001-31/4).

## Results

During the 6-month period between 30 June and 31 December 2008, 17,515 individuals aged 70 years or older entered the late life dependency period according to our definition, i.e., started to use long-term care due to need for help with PADLs (Table 1). Among these debutantes, 10,939 (62.5%) were women. At the end of the follow-up, most individuals of the study cohort were deceased (*n* = 15,515, 86.7%), corresponding to 83.2% of the women and 92.5% of the men. In the oldest age group (95 + years), 92.3% among women and 99.2% among men were deceased.

**Table 1:** Cohabitation status, level of education, country of birth and proportion that died before 31 of December 2015, among people 70 years and older who entered the late life dependency period during autumn 2008, by gender and age group

Age group	70–74	75–79	80–84	85–89	90–94	95 +	Total
	*n* = 723	*n* = 1662	*n* = 2955	*n* = 3665	*n* = 1660	*n* = 274	*n* = 10,939
Women	%	%	%	%	%	%	%
*Cohabitation status*							
Living alone	66.0	71.2	77.6	85.2	91.8	93.8	80.9
Cohabiting	34.0	28.8	22.4	14.8	8.2	6.2	19.1
*Education*							
Compulsory	49.9	56.4	62.4	65.8	69.2	73.0	63.1
Upper secondary	36.4	31.9	28.4	26.8	21.5	20.4	27.7
Tertiary	13.7	11.7	9.2	7.4	9.3	6.6	9.2
*Country of birth*							
Sweden	83.8	88.3	90.5	94.6	94.5	93.8	91.8
Nordic	8.6	7.7	6.0	3.1	3.0	2.9	4.9
Outside Nordic	7.6	4.0	3.5	2.3	2.6	3.3	3.3
Deaths	72.5	73.7	80.7	86.7	92.7	92.3	83.2

Age group	70–74	75–79	80–84	85–89	90–94	95 +	Total
	*n* = 694	*n* = 1172	*n* = 1819	*n* = 1994	*n* = 775	*n* = 122	*n* = 6576
Men	%	%	%	%	%	%	%
*Cohabitation status*							
Living alone	62.2	54.8	49.6	52.3	56.4	72.1	53.9
Cohabiting	37.7	45.2	50.4	47.7	43.6	27.9	46.1
*Education*							
Compulsory	50.6	55.9	56.0	56.8	59.3	60.7	56.1
Upper secondary	33.3	31.1	30.1	32.2	26.3	24.6	30.7
Tertiary	16.1	13.0	14.0	11.0	14.3	14.7	13.2
*Country of birth*							
Sweden	86.2	89.0	92.2	95.2	97.3	98.4	92.6
Nordic	6.8	5.5	4.1	2.4	0.7	1.6	3.7
Outside Nordic	7.1	5.5	3.7	2.4	1.9	0.0	3.7
Deaths	80.4	88.1	93.0	96.2	98.2	99.2	92.5

In general, women entered the late life dependency period at a higher age than men, with a mean age of 84.1 years compared with 83.0 years among men (not shown). Among men, the mean age at entrance was similar among those living alone (82.9 years) and those cohabiting (83.1 years). However, cohabiting female debutantes were younger (81.7 years) compared to those living alone (84.7 years).

With increasing age, both men and women were more likely to live alone, to only have compulsory education and to be born in Sweden. Men were more likely than women to cohabit (46.1% vs 19.1%) and to have higher education (compulsory education: 56.1% vs 63.1%). Among both men and women, 9 out of 10 were born in Sweden (92.6% and 91.8%).

The descriptive results (Table 2) show that entry of late life dependency at an older age was more common among women, those living alone, those with lower education and Swedish-born people. With respect to educational level, results probably reflect a cohort effect, i.e., the likelihood of having a higher education is greater in later cohorts, and hence in our younger age groups. Likewise, older debutants are more likely to live alone compared with younger debutants.

**Table 2:** Median and estimated median differences (Laplace regression) in months with late life dependency among people 70 years and older who entered the late life dependency period during autumn 2008 in Sweden

	*n*	Median age at entrance	Median no of months	Bivariate*		Adjusted†	
		into Dependency	With dependency	Coef	*P*-value	Coef	*P*-value
*Age group*							
70–74	1417	72	40.0	*ref*		*ref*	
75–79	2834	77	38.2	− 1.65	0.404	− 1.45	0.403
80–84	4774	82	34.8	− 5.01	0.008	− 4.88	0.002
85–89	5659	87	31.5	− 8.38	< 0.001	− 9.24	< 0.001
90–94	2435	91	27.1	− 12.80	< 0.001	− 14.18	< 0.001
95 +	396	96	24.3	− 15.49	< 0.001	− 17.69	< 0.001
*Gender*							
Men	6576	84	22.6	*ref*		*ref*	
Women	10,939	85	40.0	17.42	< 0.001	17.12	< 0.001
*Cohabitation status*							
Living alone	12,398	85	36.2	*ref*		*ref*	
Cohabiting	5117	83	25.7	− 10.48	< 0.001	− 5.92	< 0.001
*Education*							
Compulsory	10,594	85	32.3	*ref*		*ref*	
Upper secondary	5045	84	32.4	0.10	0.924	0.61	0.440
Tertiary	1876	83	34.3	2.00	0.132	3.80	< 0.001
*Country of birth*							
Sweden	16,132	84	32.2	*ref*		*ref*	
Nordic	781	81	38.5	6.25	0.003	3.34	0.125
Outside Nordic	602	81	35.0	2.95	0.298	0.43	0.873
Constant						31.74	< 0.001

^*^Estimates are based on Laplace regression models for each variable separate

^†^Estimates are based on a Laplace regression model adjusted for all other variables

The results further show a negative association between age at entrance and the duration of late life dependency. Median months with late life dependency decrease with each older age group. While people who *enter* late life dependency between the age of 70–74 years spent on average 40.0 (median) months with dependency before death, the corresponding number is 24.3 months for people who *entered* late life dependency at an age of 95 or older (estimated median differences in months = − 15,49; *p*-value < 0.001). The association between age at entrance and the time spent in late life dependency was robust to the adjustment of gender, cohabitation status, education and country of birth.

**Fig. 1:**
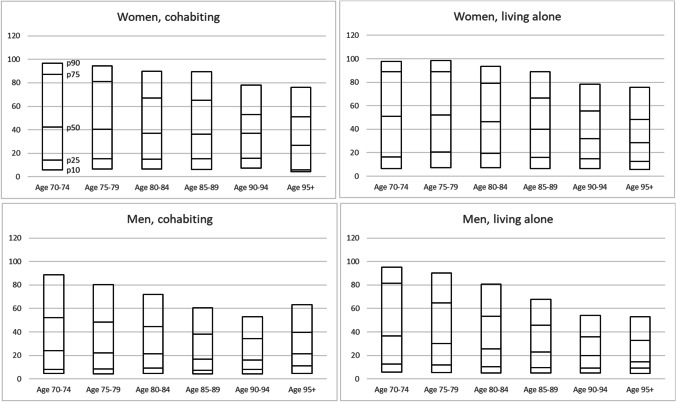
Crude percentiles (p10, p25, p50, p75 and p90) of number of months with late life dependency among people 70 years and older who entered the late life dependency period during autumn 2008, by gender, cohabitations status and age group

Finally, we calculated crude percentiles (p10, p25, p50, p75 and p90) of months with late life dependency, by age group, for women and men divided by cohabitation status (Fig. 1). The lines within the bars indicate the number of months when, e.g., 10 percent (p10) of the group had died. The results can be summarized in three main findings. First, regardless of age at entrance, the 25% individuals (debutants) who survived for the shortest time (p25) died approximately within one year among men and within 1.5 years among women. Second, the negative association between age at entrance and duration of late life dependency was clearest among those who survived longer, that is, among those still alive after the first 50% (p50) of the debutants died. Third, the negative association between age at entrance and duration of late life dependency was strongest among men who lived alone. In addition, Fig. 1 also confirms the results from Table 2, i.e., men tended to spend a shorter time with late life dependency compared to women, and cohabiting people spent a shorter time with late life dependency compared to people living alone.

## Discussion

In this large prospective study of older adults entering the period of late life dependency, measured by use of long-term care due to dependency in PADLs, we found that the majority spent a rather long period with dependency. The median number of months were 40.0 (3.3 years) for women and 22.6 (1.9 years) for men. A higher age at entry into this phase of life was associated with a shorter duration of dependency before death. This association was robust to adjustment for cohabiting at baseline, gender, education and country of birth. These results, based on a non-conventional way of capturing late life dependency, provide insights both for public health policies and for old age care planning.

For practical reasons, it is difficult to measure the length of the period with dependency in population-based studies. To our knowledge, only one study has done this, and the result showed a rather long average time spent with at least some dependencies, between 4–5 years among those who experienced severe dependency before death, but shorter in other groups, such as people without severe dependency before death and people with dementia (Romoren and Blekeseaune 2003). However, this local study was performed several decades ago and followed a small subset of the population.

We found that a higher age at entrance into late life dependency was associated with a shorter duration (i.e., the fourth age). This could be due to several reasons and factors. First, it could be that people who enter late life dependency at a higher age have managed to postpone dependency but that it deteriorates faster. It could also suggest that people who enter late life dependency at a higher age have found ways to compensate for their dependency, so they enter long-term care with higher levels of dependency than early starters. More research is needed to understand how different conditions may affect the interplay between dependency and mortality.

Another interpretation could be that the “effect” of the increased mortality risk in older age (Wang et al. 2012) reduces the effect of entering old age in good health, i.e., the increase in mortality risk by older age groups is greater than the protection from good health. We could also expect different patterns of cause of death in different age groups. In general, relatively younger decedents are more likely to die from fast-acting incurable diseases (e.g., specific types of cancer that are related to a rapid terminal decline) and older decedents are more likely to die from degenerative diseases associated with a longer period of dependency (Cohen-Mansfield et al. 2018). Still, degenerative diseases may also occur at a relatively young age and hence, people entering the period with dependency earlier in life consist of both people with fast-acting incurable diseases and those with degenerative diseases associated with a longer period with dependency.

Another potential factor behind the association between age of entry and the duration of late life dependency might be related with the threshold for entering long-term care, which has been reported to vary over time (Lagergren 2018), probably due to economic pressure on the long-term care system. During the last two decades, the supply of residential care has been reduced by 30 percent, resulting in risen eligibility thresholds for access and decreased length of stay (Schön et al. 2016). However, as our measure of late life dependency implies dependency with PADLs, a majority of individuals with such a high level of dependency has been shown to be captured by the Swedish long-term care system, either by residential care or home care (Lagergren et al. 2016). Since we included both of these main forms of long-term care in our study and used help with PADLs as a measure of late life dependency, our results are probably less sensitive for changes in the general access to long-term care.

In line with previous research (Kingston et al. 2017; Meinow et al. 2020a; Smith et al. 2013; Sundberg et al. 2021), we also found a significant gender differences in the period spent with late life dependency. Women spent almost twice as long time in late life dependency (40.0 median months) compared with men (22.6 median months), and this association could not be explained by differences in age, cohabitation status, education or country of birth.

We also found differences between cohabiting and non-cohabiting older adults. Especially among cohabiting men, the entrance into late life dependency was later and the duration was shorter compared to non-cohabiting men. This finding is probably due to (unmeasured) informal care provided by the spouse. Previous research suggests that living together with someone can postpone the use of long-term care (Meinow et al. 2005, 2020b). This is particularly the case among men, who tend to couple with younger spouses. A younger spouse is more probable to provide the support needed to delay the use of formal care. Based on the association between cohabitation status and use of long-term care, we assume that the approximation of late life dependency with use of long-term care is more reliable among people living alone.

### Limitations

The main limitation of this study is that the incidence of dependency cannot be measured by direct observations in Swedish administrative data. We used long-term care due to dependency in PADLs as a proxy measure of late life dependency. In the Swedish context, where almost all long-term care is publicly funded and needs-assessed, the specificity can be assumed to be high (e.g., the identified subjects are dependent) (Lagergren et al. 2016). However, the sensitivity of our measure is most likely lower. Mainly, the agreement between dependency and use of long-term care can be reduced by informal care. For example, if a spouse acts as caregiver, a dependent person may delay the use of long-term care (Genet et al. 2011). However, stratified analyses by cohabitation status did not alter the general results of the study. Although we acknowledge that other caregivers than spouses also provide informal care, this cannot be assessed in our data.

Another related limitation of using long-term care as a proxy for late life dependency is the difficulty to compare entrance into and duration of late life dependency between countries or over time, due to differences or changes in policies and thresholds for eligibility to long-term care. However, we tried to minimize this problem by following a cohort that entered long-term care at the same time point, that is, with the same policies and threshold for being granted long-term care. We also used a rather strict definition of late life dependency based on long-term care use due to PADL dependency to minimize the extent to which we underestimate the period with late life dependency when care needs are still restricted to practical tasks, such as household chores. In this phase of late life dependency with minor care needs, it may be more common to only rely on informal care or privately purchased help. Although the intensity of care may vary, for Sweden, it has been estimated that about 80 percent of those who need help with PADL’s use formal care to some extent (Lagergren et al. 2016), while about 20% of those who die at an age of 67 or older did not use any long-term care during their last years of life (Meinow et al. 2020a).

On the other hand, we may, to some extent, overestimate the duration of the period with late life dependency, since we miss people who entered and died within the six-month period between June and December 2008 and 31 December 2008.

The SSR is an administrative register not established for research purposes. Hence, the data include some sources of bias, such as regional differences in reporting (e.g., how the amount/time of home care is registered). To avoid the inclusion of persons with short-term needs, we restricted our analyses to persons who used long-term care for PADLs at all remaining timepoints until death (allowing for one missing time point). This was done to get more conservative estimates of the duration of late life dependency and to reduce the potential influence of erroneous reporting.

## Conclusions

This study suggests that a higher age of entrance into late life dependency is associated with a shorter period of late life dependency. Assuming that the use of long-term care in Sweden is a fairly good proxy for late life dependency, results from this study support the ambition of public health initiatives and interventions aimed at preventing or delaying entrance into of late life dependency toward higher ages.

The clear association between age at entrance and a shorter period with late life dependency could also have implications for the discussion about compression or postponement of poor health (Kingston et al. 2017; Sundberg et al. 2016). If societies manage to push the entrance into dependency to later age with the same speed that life expectancy increases, a relative compression of years with disability will occur, as the time spent with disability will consist of a relative shorter percentage of the total life span (Fries et al. 2011). This can be assumed to be desirable both from an individual and a societal perspective.

Finally, it is important to monitor the duration of dependency and long-term care use to understand the future demand of elder care. The large cohorts born in the 1940s are coming of age and age at death is still increasing. Thus, to better predict future need for long-term care, it is essential to also understand at what age and for how long older adults will need long-term care. In this study, only one cohort is followed. Future research should study cohort differences as this could provide information about whether the time with dependency is being compressed, postponement or something in between.
